# Polysilazane‐Coated Films Achieving Record‐High Moisture Barrier Performance with Sub‐10 Seconds Densification Using High‐Power VUV Irradiation

**DOI:** 10.1002/advs.202415721

**Published:** 2025-02-22

**Authors:** Luyang Song, He Sun, Yoshiyuki Suzuri

**Affiliations:** ^1^ Innovation Center for Organic Electronics (INOEL) Yamagata University Arcadia 1‐808‐48 Yonezawa Yamagata 992‐0119 Japan

**Keywords:** perhydropolysilazane (PHPS), organic and perovskite solar cells, photo‐densification, ultra‐high moisture barriers, VUV Irradiation

## Abstract

An ultra‐high moisture barrier compact SiN_X_ film can be achieved from solution‐processed perhydropolysilazane (PHPS) through vacuum ultraviolet (VUV) light exposure. This study investigates the photochemical reactions and photo‐densification of PHPS‐based barrier films under varying VUV light intensities, focusing on their effects on barrier performance. Photo‐dehydrogenation of PHPS, involving N─H and Si─H bond cleavage, is efficient and unaffected by light intensity. However, photo‐densification shows a strong dependence on light intensity, particularly above 290 mW cm^−2^. Higher intensities enhance Si─N bond cleavage, alter film dynamics, and reduce free volume through bond rearrangement, facilitating rapid network reconstruction essential for ultra‐high barrier properties. High‐power VUV light at 309 mW cm^−2^ establishes a new benchmark for ultra‐high barrier films via solution processing, achieving a record‐low average water vapor transmission rate (WVTR) of 1.6 × 10^−5^ g m^−2^ day^−1^. Films are produced in under 10 s per layer, maintaining a barrier property of 3.8 × 10^−5^ g m^−2^ day^−1^. The optimal refractive index for the top 30 nm layer is 1.74–1.77, controlling WVTR within 10^−5^ g m^−2^ day^−1^, ensuring superior barrier performance for flexible electronic devices, such as perovskite solar cells and organic photovoltaics.

## Graphical abstract

This research reveals that under high‐intensity VUV light, photo‐densification reactions of PHPS exhibit multiphoton mechanism. This enables the fabrication of SiN_X_‐based barrier films reaching record high WVTR of 1.6 × 10^−5^ g m^−2^ day^−1^ at 309 mW cm^−2^ for 6 J cm^−2^. At even lower dose of 3 J cm^−2^, single layer treatment time can be reduced to 10 s maintaining extremely low WVTR.

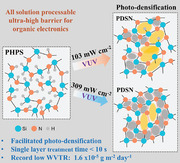

## 1 Introduction

Among next‐generation solar cells, perovskite solar cells (PSCs) and organic photovoltaics (OPVs) have received much attention.^[^ ^1^, ^2^ ^]^ They have now reached efficiency levels compared to conventional silicon‐based solar cells^[^ ^3^, ^4^ ^]^ and are on the edge of commercialization.^[^ ^5^, ^6^ ^]^ PSCs and OPVs also have two significant advantages over silicon solar cells: flexibility and solution‐processability.^[^ ^7^, ^8^, ^9^ ^]^ They allow the manufacturing of soft, flexible solar cells on plastic substrates at low temperatures. This would make greatly dynamic of solar cell installation and reduce shipping cost due to flexibility and lightweight. Furthermore, the solution process will realize mass production of high‐efficiency,^[^ ^10^ ^]^ low‐cost,^[^ ^11^ ^]^ and low‐carbon, and it will enhance the productivity of industrial production.^[^ ^12^, ^13^, ^14^ ^]^ All the features are also highly meaningful to organic light‐emitting diodes (OLEDs) and organic thin film transistors. However, PSCs and other similar devices are much more susceptible to degradation by water vapor and oxygen than the traditional inorganic semiconductors, for which water vapor is the most significant factor contributing to the degradation.^[^ ^15^, ^16^ ^]^ For flexible panels using plastic films in protection against atmospheric moisture, water vapor barrier installations are typically unavoidable.^[^ ^17^ ^]^ A multilayered barrier structure is frequently used for flexible OLEDs and is widely known as thin film encapsulation (TFE).^[^ ^18^ ^]^ Barrier performance against water vapor, often measured as water vapor transmission rate (WVTR), is crucial for the longevity and efficiency of organic electronic devices. WVTR is characterized by significantly lower values, indicating higher barrier performance. A lower WVTR means less water vapor permeates the barrier film, which is essential for protecting the sensitive organic materials from degradation caused by moisture. Basically, WVTR is required to be lower than 10^−5^ g m^−2^ day^−1^ for flexible OLED displays.^[^ ^19^, ^20^ ^]^ As to PSCs and OPVs as a consensus, the WVTR is supposed to be lower than 10^−3^ g m^−2^ day^−1^ for different applications.^[^ ^21^, ^22^, ^23^, ^24^ ^]^ The requirements are much higher compared to the performance of common barrier films. As an example, Al‐deposited films, which have WVTR performances around 0.1 g m^−2^ day^−1^. It means the performance enhancement should be more than 1000 times better expected for PSC and OLED applications. The TFE technology has already found its way into flexible OLEDs, where the multi‐layer structure is based on vacuum‐deposited silicon nitride (SiN_X_) films^[^ ^25^, ^26^, ^27^ ^]^ and typically deposited using plasma‐enhanced chemical vapor deposition (PE‐CVD)^[^ ^28^ ^]^ or sputtering^[^ ^29^ ^]^ to reach, for example, WVTR values at ≈10^−5^ g m^−2^ day^−1^. These vacuum deposition techniques have not been adapted to PSC and OPV devices, simply because they do not match the industrial requirement for mass productivity. So that, there is a strong demand for solution‐processable barriers for the next‐generation solar cells.

**Scheme 1:**
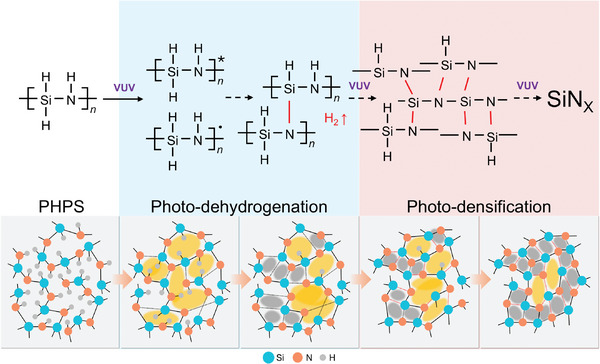
Mechanism of photo‐dehydrogenation and photo‐densification for VUV irradiated PHPS.

There are several methods for solution‐based processes to prepare barrier films.^[^ ^30^, ^31^ ^]^ Among these, even though organic polymers have been investigated as barrier materials, achieving high barrier performance is challenging due to the large free volume and thermal motion of the polymers.^[^ ^32^ ^]^ The sol–gel method for forming inorganic oxide films is another possibility; however, the resultant films are typically porous, making them unsuitable as barrier films.^[^ ^33^ ^]^ SiO_X_ films formed by thermal or photochemical oxidation of a lower organic precursor, such as perhydropolysilazane (PHPS), generally exhibit relatively high barrier properties, with WVTR values ≈10^−3^ g m^−2^ day^−1^.^[^ ^31^, ^34^ ^]^ However, there is a high demand for better performance in next‐generation solar cells. In 2022, our research group reported the formation of a dense SiN_X_ (PDSN: PHPS‐derived SiN_X_) layer by inducing the densification of PHPS at room temperature using vacuum ultraviolet (VUV) light (*λ* = 172 nm) under nitrogen. The all‐solution process gas barrier technique prevents PHPS from oxidation under nitrogen environment and promotes the formation of SiN_X_ film through dehydrogenation. A single unit of a planarization layer and PDSN layer achieved a WVTR of 2.2 × 10^−4^ g m^−2^ day^−1^, whereas a three units (6 layers) resulted in a world‐class WVTR of 4.8 × 10^−5^ g m^−2^ day^−1^.^[^ ^35^ ^]^ We previously demonstrated that the VUV exposure reaction occurs in two stages: the first being the formation of Si─N bonds via photo‐dehydrogenation of PHPS, and the second being the photo‐densification of the SiN_X_ film, as shown in **Scheme** **1**. During photo‐densification, the film composition remained constant under VUV light exposure, but the refractive index continued to increase.^[^ ^32^ ^]^ This suggests a reduction in free volume and an increase in density, both crucial for achieving superior barrier performance. Photo‐densification is slower than PDSN formation, with optimal barrier films obtained under the conditions of 10 min at 20 mW cm^−2^ and 2.4 min at 85 mW cm^−2^. Although the high‐speed coating process is industrially feasible, the relatively long light exposure time partially offsets the advantages of the solution process. Besides, no dependence on lamp power has been observed for film quality or barrier performance at lamp powers up to 85 mW cm^−2^.

In the present work, we examine the photo‐densification process of PDSN films from PHPS under VUV light as a function of light intensity. Three main goals have been defined. First, to gain a better understanding of the photochemical reaction mechanism of PHPS forming PDSN and the subsequent photo‐densification process. Second, to determine how these reactions are influenced by varying light intensities. To achieve highly efficient and dense PDSN, it is essential to understand the film formation mechanism in detail, particularly the dynamics of the photo‐densification process. This process can be significantly enhanced by generating densely excited states at high irradiance. Previous studies have not confirmed this effect with low irradiance VUV light up to 85 mW cm^−2^, so this study investigates light intensities of 100 mW cm^−2^ or higher. The third goal is to improve barrier performance and reduce process time through increased light intensity. Understanding the impact of light intensity on the performance of the PDSN barrier is crucial. If the film formation process is multi‐photonic, higher light intensity should result in a markedly shorter process time. Specifically, we are aiming to achieve a process time in the order of a few seconds, which is of great industrial value prioritizing their scalability for high‐throughput roll‐to‐roll (R2R) processes.

## 2 Results and Discussion

### 2.1 Influence of VUV Light Intensity on the Formation and Stability of PHPS‐Based Barrier Films

To elucidate the reaction mechanism of PHPS, we employed Fourier‐transform infrared spectroscopy (FTIR) to track the reduction of Si─H and N─H bonds and the formation of Si─N bonds in PHPS films under irradiation of various VUV light (Wavelength = 172 nm) powers. The PHPS films were made from its 10% dibutyl ether solution. The solution was then spin‐coated onto silicon wafers in the air and subjected to VUV irradiation at different intensities (103, 229, and 309 mW cm^−2^) under nitrogen. The films were set in a VUV irradiation apparatus with an excimer lamp placed 2 mm from the sample. The lamp power was measured using a power meter placed 2 mm from the excimer lamp, which is the same distance as in the actual use.

**Figure 1:**
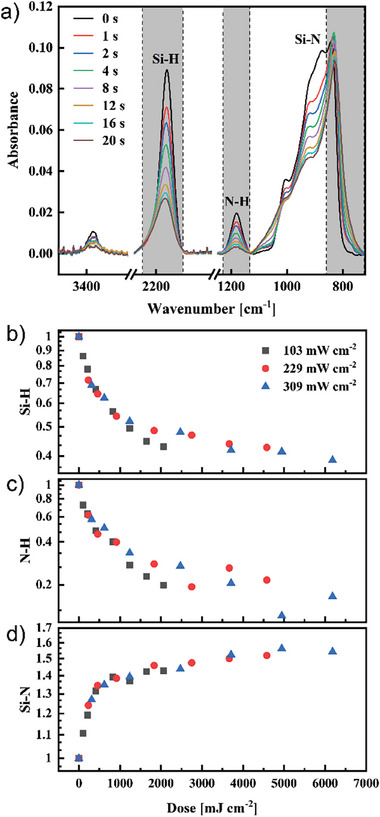
a) FTIR spectra changes of PHPS film irradiated by 103 mW cm^−2^ VUV light. Normalized change in the ratio of b) Si─H, c) N─H, and d) Si─N bonds as a function of dose amount under different VUV light intensities.

The results of the changes in the FTIR spectra of PHPS under 103 mW cm^−2^ irradiation over time are shown as an example in **Figure** **1**a. Initially, the spectrum of unirradiated PHPS (black spectrum in the figure shows N─H bond stretching and bending peaks at 3375 and 1180 cm^−1^, respectively, and the Si─H bond stretching and bending peaks are located at 2163 and 920 cm^−1^, respectively. The independent peaks for N─H and Si─H bonds were monitored at 1180 and 2163 cm^−1^, respectively. These peaks rapidly diminished immediately after VUV irradiation, indicating the progression of the formation to Si─N bonds. On the other hand, the initial broad Si─N peak, ranging from 800 to 1000 cm^−1^, attributed to various components, shifted toward lower wavenumbers with VUV irradiation. This shift suggests the formation of SiN_X_‐like films. Due to the complexity of fitting Si─N peaks, we estimated the peak area within the range where the differential spectra appear continuously changing, as shown in Figures S1–S3 (Supporting Information).

Figure 1b–d illustrates the variations in Si─H, N─H, and Si─N bonds as a function of irradiation dose under different light intensities. In Figure 1b,c, it is observed that both Si─H and N─H bonds decrease with increasing dose, eventually plateauing. Correspondingly, Figure 1d shows that the peak area of Si─N bonds increases with dose. These changes confirm the photo‐dehydrogenation reaction of Si─H and N─H bonds under VUV irradiation (as depicted in Scheme 1), leading to the formation of new Si─N bonds. Based on the FTIR results shown in Figure 1b–d, the concentration changes of the reactants (Si─H and N─H) and the product (Si─N) exhibit a segmented linear relationship with the dose. Unlike homogeneous reactions in solution, the reaction here occurs in solid films, where the availability and diffusion of reactants are inherently restricted. In this scenario, Si─H bonds cannot readily reach nearby unexcited N─H bonds, leading to a gradual decline in the reaction rate rather than the exponential decay typical of first‐order solution processes. This limitation gives rise to a second region in the segmented relationship, where the reaction rate slows down. Besides, the VUV absorption of generated Si─N is higher than that of PHPS, which was claimed in our previous work.^[^ ^35^ ^]^ So, as the SiN_X_ layer forms on top, it acts as a filter, reducing the amount of VUV light that penetrates deeper into the film. This attenuation leads to a lower photon flux reaching the interior regions of the film, contributing to the observed decrease in reaction rate over longer irradiation times as well. Additionally, in the original PHPS, the Si/N ratio is ≈1:1, where Si can form four bonds while N can form only three. This leads to an insufficiency of N─H bonds. This also contributes to the trend of the reaction reaching a plateau. It has been reported in the past that residual hydrogen also affects the barrier performance of SiN_X_ films fabricated by the CVD method.^[^ ^36^, ^37^, ^38^ ^]^ Therefore, addressing this issue is crucial. Interestingly, as shown in Figure S4 (Supporting Information), while higher light intensity accelerates the generation of Si─N bonds per unit time (shortening the overall reaction time), it does not fundamentally change the reaction per photon absorbed. At equivalent dose levels, as shown in Figure 1b–d, the extent of reaction and the amount of Si─N bonds formed remain consistent across varying light intensities. This confirms that the photo‐dehydrogenation reaction is dose‐dependent and intensity‐independent, indicating that the initial photo‐dehydrogenation reaction in PHPS is predominantly a single‐photon process. Due to the rapid nature of the photochemical reaction, the surface reaction initially transitions from dehydrogenation to photo‐densification. During photo‐densification, Si─N bonds are cleaved and reformed with nearby broken bonds, spontaneously rearranging to optimize the film structure and reduce the free volume of film (Scheme 1). This allows unreacted Si─H and N─H bonds another opportunity to form new Si─N bonds. Therefore, in the later stages, the formation of Si─N bonds does not cease completely but shows a slight increase. The process of photo‐densification will be discussed in detail in the next section.

The candidates for the reactive species are the excited states of PHPS or radical species that have been dehydrogenated from the excited states, as shown in Scheme 1. L. Prager and his coworkers had studied based on quantum‐chemical calculations, which revealed that excitation by high‐energy VUV photons results in the interconversion of PHPS to the S_1_ singlet state, intersystem crossing to the T_1_ triplet state, and the subsequent Si─N bond scission may happen to achieve radical species.^[^ ^39^ ^]^ Even though, the present experiment showed that there is no acceleration effect of light intensity and that the dose amount determines the reaction amount regardless of lamp power. In the process of dehydrogenation of PHPS by VUV light and formation of new Si─N bonds, there is no acceleration of the reaction with respect to light intensity, and the time to obtain the same reaction amount is inversely proportional to the light intensity under conditions with sufficient amounts of Si─H and N─H. It reveals that the photo‐dehydrogenation reaction obeys a single photon reaction mechanism.

### 2.2 Characterization of PHPS Film Densification Process

**Figure 2:**
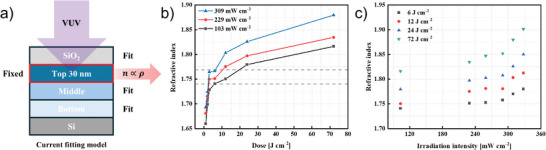
a) Fitting model of PDSN layers. b) Refractive index of the Top30 nm as a function of dose. c) Relationship between refractive index and irradiation intensity (103–328 mW cm^−2^).

The refractive index of the obtained films was evaluated from spectroscopic ellipsometry. Spectroscopic ellipsometry can determine the physical parameters of thin films such as thickness, refractive index, and extinction coefficient. It is known that in thin films with little compositional change, density correlates with refractive index. For example, the density of silicon nitride layers with the same composition has a linear relationship with their refractive index.^[^ ^40^, ^41^, ^42^, ^43^, ^44^, ^45^ ^]^ Therefore, in this study, the refractive index can also be used to evaluate the densification level of the VUV‐irradiated PHPS films. Our previous work reported that VUV irradiation results in a refractive index gradient across the film thickness due to the high absorption coefficient of the resulting PDSN films under 172 nm VUV irradiation.^[^ ^32^, ^39^, ^46^, ^47^ ^]^ This leads to a higher refractive index (higher density) close to the surface exposed to VUV light. In this study, a four‐layer model consisting of one SiO_2_ layer and three PHPS layers (Top, Middle, Bottom) was applied as shown in **Figure** **2**a. The mean squared error (MSE) for all the fittings was below 4, indicating the appropriateness of our model. The experimental and fitting results from the ellipsometry measurement are shown in Figures S5 and S6 (Supporting Information), and their refractive index distributions are summarized in Figure S7 (Supporting Information). As the topmost layer receives the most light, it leads to a higher refractive index, which means more effective photo‐densification. Therefore, we fixed the Top layer with the highest refractive index at 30 nm (Top30 nm) to reveal the details of the photo‐densification process with light intensity (Figure 2b). While the refractive index likely changes continuously due to varying VUV light penetration, our fixed thickness approach helps highlight the influence of light intensity. Previous studies by Sasaki et al. fitted a six‐layer model to PDSN films, identifying the densest layer with a refractive index of 2.09 at depths between 25 and 40 nm. This suggests a refractive index gradient even within the 30 nm Top layer, supporting our approach to using average density for discussions. This Top30 nm could be the densest layer in the PDSN film and thus acts as a substantial barrier layer. Despite the same dose, it was observed that as the light intensity increases, the refractive index of Top30 nm also increases, following the trend n(309 mW cm^−2^) > n(229 mW cm^−2^) > n(103 mW cm^−2^). If photo‐densification process follows a single photon reaction kinetics, where it is the case of photo‐dehydrogenation, the same amount of dose should yield the same refractive index, irrespective of the light intensity. Regarding the literature, it has been documented that Si─N bonds can be cleaved by VUV irradiation.^[^ ^39^, ^48^ ^]^ Under an inert atmosphere, the resulting Si or N species must react or recombine with nearby atoms, as no external reactants are involved. Therefore, we have considered that in the photo‐densification process, the free volume inside the film decreases due to the repeated cleavage and rearrangement of the Si─N bonds by VUV light, which is the presumed mechanism of photo‐densification described in Scheme 1. In this study, our findings first demonstrate that the lamp power significantly impacts this process beyond 100 mW cm^−2^, especially 290 mW cm^−2^.

The change in the average refractive index of Top30 nm against dose amount is plotted for each light intensity in Figure 2b. It has already been mentioned that the PHPS to Si─N bond formation reaction is almost completed within the first few seconds of the reaction from Figure 1d. The amount of dose at which the initial Si─N bond formation reaction is almost finished is ≈2 J cm^−2^. Therefore, this rapid increase up to a dose of ≈2 J cm^−2^ corresponds to the initial dehydrogenation in PHPS during the PHPS‐to‐PDSN conversion. Beyond this dose, the refractive index continues to increase gradually, where there is almost no change in FTIR, suggesting that a photo‐densification process occurs to rearrange the chemical structure of PDSN. It has already been mentioned that the formation of Si─N bonds during the PHPS‐to‐PDSN conversion occurs rapidly, with the majority of the reaction completing within the first few seconds, after which the reaction slows down significantly. A detailed analysis of the saturation dose for Si─N bond formation, as plotted in Figure S8 (Supporting Information), indicates that the process saturates at ≈24 J cm⁻^2^. Beyond this dose, no additional Si─N bonds are formed. The rapid increase in Si─N bonds at the beginning corresponds to the initial dehydrogenation of PHPS during its conversion to PDSN. Interestingly, the refractive index continues to increase even after the formation of Si─N bonds has plateaued, indicating no further changes in chemical composition. This observation suggests that, even if the photo‐dehydrogenation process is complete, a subsequent photo‐densification process takes place. During this phase, the PDSN undergoes structural rearrangement, resulting in a higher‐density film. The influence of light intensity is further detailed in Figure 2c. It shows the refractive index of Top30 nm against light intensity at doses of 6, 12, 24, and 72 J cm^−2^. The refractive index increases with higher light intensity but displays nonlinear characteristics. Here, if there is no acceleration effect between lamp power and refractive index, then the same amount of dose should give the same refractive index regardless of lamp power. However, the fact is, at the same dose applied, PDSN prepared at higher light intensity ends up with higher refractive index. For example, at a dose of 6 J cm^−2^, the refractive index ranges from 1.74 (103 mW cm^−2^) to 1.78 (328 mW cm^−2^). At 12 J cm^−2^, the refractive index increases from 1.75 to 1.81. At higher doses of 24 and 72 J cm^−2^, the highest refractive index reaches 1.85 and 1.90, respectively, at 328 mW cm^−2^. While the influence of light intensity is not too obvious up to 290 mW cm^−2^, exceeding this threshold results in dramatic increases in refractive index. This suggests a critical intensity above which advanced rearrangement reactions occur, accelerating the densification process. Although the refractive index is not directly related to the number of Si─N bond cleavages, the significant changes observed at high intensities indicate the importance of light intensity in the photo‐densification process. Higher light intensities facilitate more Si─N bond cleavages per unit volume, thus enhancing the dynamics of film rearrangement dramatically. It helps promote free volume reduction and contributes to obtaining a more compact film structure. Using the VUV light with an intensity of 328 mW cm^−2^, significant densification is achieved, and higher intensities are expected to further enhance this effect. This implies that higher intensity of VUV light can significantly shorten the processing time needed to achieve the same refractive index. More importantly, because of this draft increasing behavior, it reveals that the mechanism of photo‐densification process is beyond a single photon reaction such as the previous photo‐dehydrogenation reaction.

### 2.3 Evaluation of Water Vapor Permeability in High‐Intensity VUV‐Irradiated Barriers

To evaluate the densification and barrier performance of PDSN films created under different light intensities, these films were prepared on plastic substrates, and then their WVTR were measured. While the densification of the barrier film is a key factor in enhancing barrier performance, other factors such as surface roughness and particles, pinholes and cracks can also significantly affect the performance. To minimize these factors, PI film with a minimal number of particles and surface roughness was employed as the substrate, and a multi‐layered barrier structure was applied. Previous studies have shown that the WVTR of standalone PI film and PI/PDMS film are 9.0 and 1.0 g m^−2^ day^−1^, respectively, indicating that neither of them contributes significantly to barrier properties.^[^ ^35^ ^]^ To accurately assess the light intensity dependency of PDSN, and to minimize the influence of defects, we constructed an alternating layer structure of barrier layers (PDSN) and planarization layers (PDMS). The barrier film structure can be described as follows from the bottom: PI substrate / PDMS / PDSN / PDMS / PDSN / PDMS / PDSN, where one unit consists of PDMS (130 nm) and PDSN (200 nm). It ends up with forming three units (total six layers, 990 nm) on the PI film. We intentionally separated the coating and irradiation processes to align with the practical requirements of scalable production. The spin‐coating process was conducted in ambient air, as PHPS exhibits a slow oxidation rate under these conditions, and VUV irradiation was performed under nitrogen flow. The spin coater and VUV irradiation instruments (excimer lamp) were located in a HEHEPA‐filtered lean environment to minimize particle contamination. As shown in Figure S9 (Supporting Information), the obtained films comprising three SiN_X_ layers and three PDMS layers and subjected to VUV treatment, exhibit minimal curvature in the images provided. This observation suggests that the internal stress generated during the fabrication process is negligible. The WVTR of the resulting barrier films was measured using the Moresco Super‐Detect system, which employs a differential pressure method (MA method) with a MASS detector. The barrier films were fabricated in a size of 50 by 50 mm, with a measurement diameter of 40 mm. WVTR measurements were conducted under standard conditions for barrier films of 40 °C and 90% relative humidity (RH).

**Figure 3:**
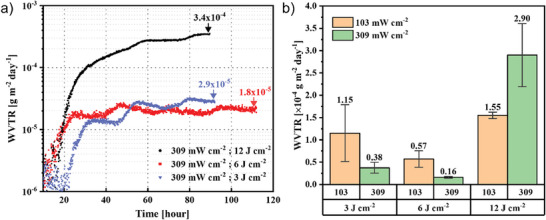
a) WVTR measurement data for barriers fabricated under different cumulative doses at 309 mW cm^−2^. b) Summary of average WVTR values at different dosing times and VUV light intensities.

**Figure** **3**a shows the WVTR measurement results for barriers irradiated under different cumulative doses under 309 mW cm^−2^ light intensities as the example, the ones under 103 mW cm^−2^ are shown in Figure S10 (Supporting Information). It can be found that in cases of 3 and 6 J cm^−2^, the WVTR values reaching equilibrium are extremely low, indicating an outstanding barrier performance. The fluctuation in the WVTR curve is caused by the unavoidable daily temperature shift, it's not substantive. Their comparisons and averages to those at 103 mW cm^−2^ are summarized in Figure 3b. Each test was conducted at least twice, with barrier films prepared under identical conditions but on different days to validate the reproducibility of the experiments. Regardless of whether the lamp power was 103 or 309 mW cm^−2^, the lowest WVTR, indicating the highest barrier performance, was achieved at a dose of 6 J cm^−2^. Specifically, under the high intensity of 309 mW cm^−2^, a dose of 6 J cm^−2^ resulted in an outstanding average WVTR of 1.6 × 10^−5^ g m^−2^ day^−1^, representing the record of highest performance for wet‐processed barrier films, a detailed comparison of barrier performance using different materials are listed in Table S1 (Supporting Information). It is nearly tripling the previously reported record barrier performance of 4.8 × 10^−5^ g m^−2^ day^−1^. Notably, even at a dose of only 3 J cm^−2^ under 309 mW cm^−2^, a high performance of 3.8 × 10^−5^ g m^−2^ day^−1^ can be achieved. The single layer irradiation time for PDSN layer at doses of 3 and 6 J cm^−2^ under 309 mW cm^−2^ are ≈10 and 20 s, respectively. It significantly shortens the light exposure process and approaches a throughput far superior to vacuum processes.

**Figure 4:**
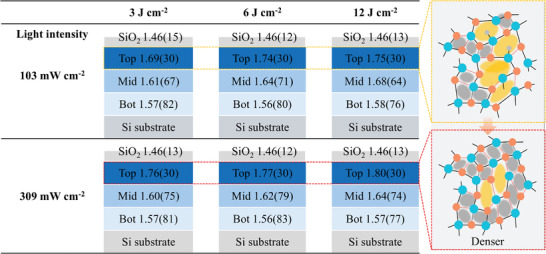
Refractive index distributions of PDSN layers irradiated with VUV light at intensities ranging from 103 to 309 mW cm^−2^ under varying doses. The refractive index of each layer is indicated along with its corresponding thickness (in brackets).

To get the idea of proper refractive index range for achieving high barrier properties, the ellipsometry results are summarized in **Figure** **4** for the PDSN layers under various lamp powers (103 and 309 mW cm^−2^) and doses (3, 6, and 12 J cm^−2^). At 309 mW cm^−2^, the refractive index of the Top30 nm was consistently higher across all conditions compared to 103 mW cm^−2^. Interestingly, a higher top layer refractive index of 1.76 was yielded at 309 mW cm^−2^ for 3 J cm^−2^, which is even higher than the one of 1.74 obtained at 103 mW cm^−2^ for higher dose of 6 J cm^−2^. It indicates a more effective film rearrangement process to achieve more compact layer under higher light intensity despite a lower dose. Consequently, the barrier performance under 309 mW cm^−2^ and 3 J cm^−2^ (WVTR = 3.8 × 10^−5^ g m^−2^ day^−1^) was ≈1.5 times as good as the one prepared under 103 mW cm^−2^ in a larger dose of 6 J cm^−2^ (WVTR = 5.7 × 10^−5^ g m^−2^ day^−1^).

**Figure 5:**
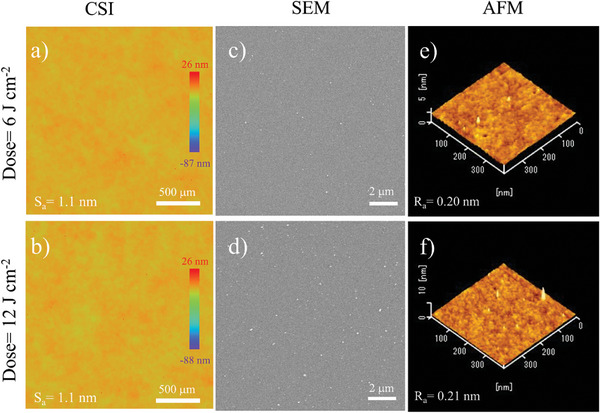
CSI, SEM, and AFM images depicting the surface morphology of barrier films fabricated under VUV irradiation at an intensity of 309 mW cm^−2^, with two different dose amounts: a), c), and e) correspond to 6 J cm^−2^, while b), d), and f) correspond to 12 J cm^−2^.

Our study demonstrates that an increasing light intensity accelerates densification, achieving world‐class barrier performance with extremely short irradiation times. However, at a high dose of 12 J cm^−2^, the barrier performance deteriorated despite an increase in refractive index observed from ellipsometry, in both cases of 103 and 309 mW cm^−2^. In principle, the densification of PHPS films is expected to improve with increasing dose, leading to better barrier performance. This trend is evident in Figure 3b for doses increasing from 3 J cm^−2^ to 6 J cm^−2^, where films prepared under higher light intensity exhibit lower WVTR values. However, at a dose of 12 J cm^−2^, the trend reverses: WVTR increases for both low‐ and high‐intensity samples, with more significant degradation in the case of high‐intensity. Previous studies suggest that excessive doses may accelerate the densification process to the point where rapid molecular rearrangement outpaces the relaxation of internal stress, leading to nanoscale cracks or other structural defects.^[^ ^35^ ^]^ Higher light intensities accelerate this densification process, promoting earlier defect formation compared to lower intensities. Consequently, films prepared at 309 mW cm^−2^ are likely to develop more defects, which accounts for the unexpected increase in WVTR performance at 12 J cm^−2^. Given that the photoreaction from PHPS to PDSN involves dehydrogenation and subsequent densification, coherence scanning interferometry (CSI), scanning electron microscopy (SEM), and atomic force microscopy (AFM) are utilized to examine the surface morphologies of PHPS films irradiated at the doses of 6 and 12 J cm^−2^ under 309 mW cm^−2^ VUV intensity. These observations provide insights into the surface characteristics at different scales (**Figure** **5**). The largest‐scale observations, obtained using CSI with a field of view of 2200 µm × 2200 µm (Figure 5a,b), revealed no noticeable differences between surfaces irradiated for 6 and 12 J cm^−2^. Both surfaces were exceptionally smooth, with surface roughness (Sa) measured at 1.1 nm. Similarly, AFM surface roughness measurements over a smaller field of view (400 nm × 400 nm, Figure 5e,f) showed uniform and flat surfaces with negligible differences average roughness (Ra): Ra = 0.20 nm (6 J cm^−2^) and Ra = 0.21 nm (12 J cm^−2^). SEM images (Figure 5c,d) also displayed no significant differences between the two conditions, apart from a slightly increased number of white spots in the 12 J cm^−2^ case. Although the composition of these white spots was not directly analyzed, a recent simulation study suggests that such features may result from the formation of Si clusters under continuous VUV irradiation.^[^ ^49^ ^]^ These clusters likely precipitate on the surface, while the size of them are very small (≈5 nm) and the underlying SiN_X_ layer remains dense and structurally intact. In fact, similar spots were also present under the optimal condition of 6 J cm^−2^, where excellent WVTR performance was achieved. Therefore, these white spots are not considered a direct reason for the barrier performance degradation observed at 12 J cm^−2^. We hypothesize that nanoscale cracks, induced by volume shrinkage during the photo‐densification process, may contribute to the observed decline in barrier performance at higher doses.

For these reasons, the appropriate refractive index of the top layer should be in the range of 1.74–1.77. This range provides important guidance as it is a key parameter for obtaining excellent barrier performance in PHPS‐based densification processes. To further enhance barrier performance and achieve WVTR = 10^−6^ g m^−2^ day^−1^, it is crucial to suppress these predicted nano‐cracks. While volume shrinkage due to dehydrogenation during photoreaction poses a fundamental challenge, the formation of nano‐defects can also be highly dependent on the thickness of barrier layer and adhesion to the underlying layer. Future improvements in layer structure, film thickness, VUV conditions, and adhesion between layers could lead to further performance enhancements.

Finally, the reduction of VUV irradiation time through increased light intensity was discussed. Previous work reported a WVTR of 4.8 × 10^−5^ g m^−2^ day^−1^ using a 3‐unit (6‐layer) barrier structure under 85 mW cm^−2^ light intensity with a dose of 12 J cm^−2^, requiring 141 s of irradiation for each PHPS layer. In this study, a light intensity of 309 mW cm^−2^ was used, which is 3.6 times higher. The detailed parameters and results are summarized in **Table** **1**. The predicted completion time was ≈40 s, even without considering accelerated densification effects. By utilizing accelerated photo‐densification, the dose was reduced to 6 J cm^−2^, achieving a WVTR of 1.6 × 10^−5^ g m^−2^ day^−1^ with an irradiation time of 20 s per layer, which is only 13% of the previous duration and 50% of the does amount. Remarkably, a dose of 3 J cm^−2^ achieved a WVTR of 3.8 × 10^−5^ g m^−2^ day^−1^ with astonishingly low duration of irradiation per layer within 10 s, reducing the time to 6% of the previous duration and 25% of the applied dose. These results show the strong acceleration effect of high‐intensity irradiation in photo‐densification. High‐intensity light helps create higher density films with lower doses. This acceleration effect is likely due to the increase in the number of Si─N bonds breaking and reforming during film rearrangement, which improves film dynamics and increases density.

**Table 1:** Summary of refractive index, WVTR, and single‐layer irradiation time parameters for barrier films prepared at VUV intensities of 103 and 309 mW cm⁻^2^ under varying doses.

Lamp power		3 J cm^−2^	6 J cm^−2^	12 J cm^−2^
103 mW cm^−2^	The refractive index of top layer	1.69	1.74	1.75
	WVTR (g m^−2^ day^−1^)	1.2×10^−4^	5.7×10^−5^	1.6×10^−4^
	Irradiation time(s)	30	60	120
309 mW cm^−2^	The refractive index of top layer	1.76	1.77	1.80
	WVTR (g m^−2^ day^−1^)	3.8×10^−5^	1.6×10^−5^	2.9×10^−4^
	Irradiation time(s)	10	20	40

Despite achieving high barrier performance in this study, it still has potential for making further improvements. Various parameters in the barrier fabrication process require optimization and deeper investigation. In particular, interfacial stress plays an important role in determining the performance of multi‐layered encapsulations. Stress‐related defects, such as cracks and delamination, can result from three main factors: bulk stress, surface adhesion, and film thickness. Bulk stress, primarily caused by dehydrogenation during VUV irradiation, is inherent and difficult to control. In this study, UV‐curable PDMS (Shin‐Etsu Chemical) was used as a stress–relief and planarization layer between PHPS layers to mitigate bulk stress. Exploring alternative intermediate materials may help with further improvements. Surface adhesion, another crucial factor, directly impacts on the integrity of the multi‐layer structure. PHPS can react with surface ─OH groups, which enhances adhesion. In our work, VUV treatment of the PDMS surface converted it to SiO_X_, introducing ─OH groups and reducing the water contact angle from 107° to 15°, thus improving adhesion with PHPS. Future advancements in surface treatments and alternative materials are expected to further address delamination and cracking issues. Film thickness also influences stress and barrier performance. Thinner films inherently generate less stress, reducing the possibility of interfacial failure. However, thinner films may compromise the barrier properties. In this study, a 200 nm PHPS film thickness was employed based on prior research, but future investigations will explore optimization of thickness across varying light intensities to achieve a better balance between stress mitigation and barrier performance.

## 3 Conclusion

This study comprehensively investigates the photochemical reactions and photo‐densification processes of PHPS‐based barrier films under varying VUV intensities, and their barrier performance. Initially, the photo‐dehydrogenation reactions in PHPS, characterized by the cleavage of N─H and Si─H bonds, are analyzed as highly efficient single‐photon reactions, where light intensity does not accelerate the reaction rate. However, during the photo‐densification process, a significant dependence on lamp power is observed, especially at intensities above 290 mW cm^−2^, where an acceleration effect becomes obvious. Higher light intensity increases the simultaneous cleavage of Si─N bonds, altering film dynamics and reducing free volume through bond rearrangement. High‐intensity light significantly impacts the densification process, promoting rapid and extensive bond cleavage and network reconstruction, which are essential for achieving ultra‐high barrier properties. Remarkably, the application of high‐power 309 mW cm^−2^ VUV light has set a new standard in the production of ultra‐high barrier films via solution processing. Achieving a record‐low average WVTR of 1.6 × 10^−5^ g m^−2^ day^−1^ highlights the substantial impact of high light intensity on the effectiveness and quality of these barrier films. Furthermore, the ability to achieve such high densified films at low doses represents a significant improvement. Utilizing high‐intensity VUV light enables the fabrication of high‐density barrier films in extraordinary short durations, under 10 s per layer, while maintaining a high barrier property of 3.8 × 10^−5^ g m^−2^ day^−1^. The experiment also suggests an optimal range for the refractive index of the Top30 nm layer. When this index is between 1.74 and 1.77, WVTR can be controlled within the order of 10^−5^ g m^−2^ day^−1^. This capability not only demonstrates the technical feasibility of rapid manufacturing but also ensures superior barrier performance. This is crucial for the longevity and reliability of flexible electronic devices, such as PSCs and OPVs.

## 4 Experimental Section

### Materials and Preparations

VUV irradiation and PHPS solution preparation were performed under N_2_ environmental. PDMS solution preparation, spin‐coating, and UV irradiation were performed under the air environment in HEPA filtered clean booth (25 °C, 30% RH). The precursors of UV‐curable PDMS (KER‐4690‐A and B, Shin‐Etsu chemical) were diluted 16 times with cyclopentasiloxane (KF‐995, Shin‐Etsu Chemical) (A:B:cyclopentasiloxane = 1:1:16), achieving a viscosity of less than 20 mPa s, which allows it to spread homogeneously on the substrate during the spin‐coating process. The solution was spin‐coated on the Si (100) substrates at 2000 rpm for 30s using a spin coater (MS‐B150, Mikasa). When using PI films (Xenomax, Ra: ≈0.5 nm, thickness: 38 µm, sample size: 50 mm × 50 mm, Xenomax‐Japan) as the substrate, an adsorption pad (XF0205DA, Nitto Denko) was utilized, which provides a relatively flat surface without using any adhesive in contact with PI substrate. The adhesive side of the adsorption pad was fixed onto a 5 cm × 5 cm sized glass substrate. The protective film on the back of the PI film was removed, and the exposed side of the PI film was placed evenly on the pad. The PI film self‐adhered to the pad through the displacement of air, creating a flat surface. The protective film on the front side of the PI film was then removed, and the surface underwent VUV pretreatment (172 nm wavelength, 309 mW cm^−2^, 5.1 J cm^−2^) under a 1% oxygen/99% nitrogen atmosphere. The PDMS solution was then spin‐coated onto the pretreated PI surface at 6000 rpm for 30 s. The successful formation of a continuous ultrathin PDMS film relies heavily on the wettability between the lower layer (substrate or PHPS) and the PDMS/D5 solution. For both the underlying layer of PI film and PHPS surface, VUV treatment was carefully applied to ensure their wettability. In principle, it is better to direct measure the contact angle of PDMS itself, but due to the complicity of handle PDMS solution, the contact angle of D5 was measured, which is the dilutor of PDMS and has similar structure, on top of pretreated PI film and as prepared PHPS layer, and the contact angle was 7.2° and 6.4°, respectively, indicating excellent wettability. This high affinity ensures that the PDMS spreads uniformly, maintaining a continuous thin layer before UV curing. The PDMS layers were exposed to UV light (UV‐100 high‐pressure mercury lamp, λ: 365 nm, intensity to the sample surfaces: 20 mW cm^−2^, UV dose: 2400 mJ cm^−2^, ORC Manufacturing Co.) to obtain solid PDMS layers and VUV light (λ: 172 nm, photon energy: 7.2 eV = 696 kJ mol^−1^, intensity to the sample surfaces: 309 mW cm^−2^, VUV dose: 6000 mJ cm^−2^, M.D.COM) to convert the surfaces into SiO_X_. The 10 wt% PHPS solution was prepared by diluting a 20 wt% PHPS solution (in DBE solvent, without catalysts, Shin‐Etsu Chemical) with anhydrous DBE (99.3%, Sigma–Aldrich). The solution was spin‐coated on PI/PDMS samples or Si (100) substrates at 2000 rpm for 30 s. Then, the films were exposed to VUV light (*λ*: 172 nm, photon energy: 7.2 eV = 696 kJ mol^−1^, M.D.COM) to apply the photochemical and photo‐densification reaction. Intensity to the sample surfaces were variated from 103 to 328 mW cm^−2^ (M.D.COM). The doses were controlled by varying the lamp irradiation intensities and time from 0 to 72 J cm^−2^.

### Characterization

To obtain the refractive index (n at 850 nm) and layer thickness, variable angle spectroscopic ellipsometry (VASE) measurements were conducted using a VASE 32 spectroscopic ellipsometer (J.A.Woollam). The angles of incident light ranged from 45° to 75° in steps of 5°. The experimental ellipsometry parameters (Ψ and Δ) of the PDSN layers were analyzed using a four‐layer optical model containing a SiO_2_ and three single‐layer models with Gaussian oscillators. FTIR transmission spectra were recorded using a Nicolet iS5 spectrophotometer (Thermo Fisher) in the range 500–4000 cm^−1^. Spectra were obtained using 32 scan summations at 2 cm^−1^ resolution. The WVTR was measured by the modified differential pressure method with an attached support (MA) method on a Super‐Detect (effective permeation area: 40 mmΦ, MORESCO) at 40 °C and 90% RH. This method is characterized by the use of a support layer between the space connected to the water vapor/gas Quadrupole Mass Spectrometer (QMS) and the measurement sample. So that a high degree of vacuum is always maintained in the space on the QMS side, making it possible to reach a high sensitivity of 10^−7^ g m^−2^ day^−1^ or less, with a shorter time than conventional methods.

## Conflict of Interest

The authors declare no conflict of interest.

## Supporting information



Supporting Information

## Data Availability

Research data are not shared.
