# Supplementary material for: Polysilazane‐Coated Films Achieving Record‐High Moisture Barrier Performance with Sub‐10 Seconds Densification Using High‐Power VUV Irradiation
Source: Adv Sci (Weinh). 2025 Feb 22;12(17):2415721. doi: 10.1002/advs.202415721 (PMC12061255; doi:10.1002/advs.202415721)
Supplement: Supplementary file 1 — Supporting Information [file ADVS-12-2415721-s001.docx]

Supporting Information

**Polysilazane Coated Films Achieving Record-High Moisture Barrier Performance with Sub-10 Seconds Densification Using High-Power VUV Irradiation**

Luyang Song, He Sun*, Yoshiyuki Suzuri*

*Innovation Center for Organic Electronics (INOEL), Yamagata University, Arcadia 1-808-48, Yonezawa, Yamagata 992-0119, Japan*

*E-mail: sunhepeter@yz.yamagata-u.ac.jp; suzuri@yz.yamagata-u.ac.jp*

1. **Preparation of PDMS / PHPS**

VUV irradiation and PHPS solution preparation were performed under N_2_ environmental. PDMS solution preparation, spin-coating, and UV irradiation were performed under the air environment in HEPA filtered clean booth (25°C, 30%RH). The precursors of UV-curable PDMS (KER-4690-A and B, Shin-Etsu chemical) were diluted 16 times with cyclopentasiloxane (KF-995, Shin-Etsu Chemical) (A:B:cyclopentasiloxane = 1:1:16). The solution was spin-coated on the Si (100) substrates at 2000 rpm for 30s using a spin coater (MS-B150, Mikasa). When using PI films (Xenomax, Ra: ≈0.5 nm, thickness: 38 μm, sample size: 50 mm × 50 mm, Xenomax-Japan) as the substrate, we utilized an adsorption pad (XF0205DA, Nitto Denko), which provides a relatively flat surface without using any adhesive in contact with PI substrate. The adhesive side of the adsorption pad was fixed onto a 5 cm x 5 cm sized glass substrate. The protective film on the back of the PI film was removed, and the exposed side of the PI film was placed evenly on the pad. The PI film self-adhered to the pad through the displacement of air, creating a flat surface. The protective film on the front side of the PI film was then removed, and the surface underwent VUV pretreatment (172 nm wavelength, 309 mW cm^−2^, 5.1 J cm^−2^) under a 1% oxygen/99% nitrogen atmosphere. The PDMS solution was then spin-coated onto the pretreated PI surface at 6000 rpm for 30 s. The PDMS layers were exposed to UV light (UV-100 high-pressure mercury lamp, λ: 365 nm, 254 nm, intensity to the sample surfaces: 20 mW cm^−2^, UV dose: 2400 mJ cm^−2^, ORC Manufacturing Co.) to obtain solid PDMS layers and VUV light (λ: 172 nm, photon energy: 7.2 eV = 696 kJ mol^−1^, intensity to the sample surfaces: 309 mW cm^−2^, VUV dose: 6000 mJ cm^−2^, M.D.COM) to convert the surfaces into SiO_X_. The 10 wt% PHPS solution was prepared by diluting a 20 wt% PHPS solution (in DBE solvent, without catalysts, Shin-Etsu Chemical) with anhydrous DBE (99.3%, Sigma-Aldrich). The solution was spin-coated on PI/PDMS samples or Si (100) substrates at 2000 rpm for 30s. Then the films were exposed to VUV light (λ: 172 nm, photon energy: 7.2 eV = 696 kJ mol^−1^, M.D.COM) to apply the photochemical and photo-densification reaction. Intensity to the sample surfaces were variated from 103 to 328 mW cm^−2^ (M.D.COM). The doses were controlled by varying the lamp irradiation intensities and time from 0 to 72 J cm^-2^.

1. **Characterization**

To obtain the refractive index (n at 850 nm) and layer thickness, variable angle spectroscopic ellipsometry (VASE) measurements were conducted using a VASE 32 spectroscopic ellipsometer (J.A.Woollam). The angles of incident light ranged from 45° to 75° in steps of 5°. The experimental ellipsometry parameters (Ψ and Δ) of the PDSN layers were analyzed using a four-layer optical model containing a SiO_2_ and three single-layer models with Gaussian oscillators. FTIR transmission spectra were recorded using a Nicolet iS5 spectrophotometer (Thermo Fisher) in the range 5004000 cm^–1^. Spectra were obtained using 32 scan summations at 2 cm^−1^ resolution. The WVTR was measured by the modified differential pressure method with an attached support (MA) method on a Super-Detect (effective permeation area: 40 mmΦ, MORESCO) at 40 °C and 90% relative humidity. This method is characterized by the use of a support layer between the space connected to the water vapor/gas Quadrupole Mass Spectrometer (QMS) and the measurement sample. So that a high degree of vacuum is always maintained in the space on the QMS side, making it possible to reach a high sensitivity of 10^−7^ g m^−2^ day^−1^ or less, with a shorter time than conventional methods.


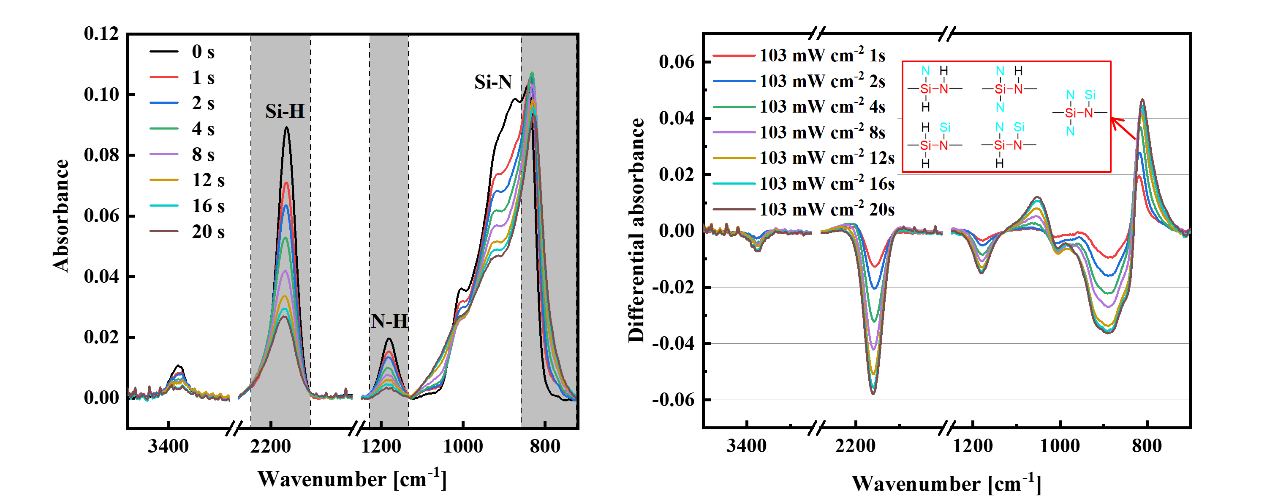


**Figure S1.** FTIR spectra changes (left) and differential spectra (right) of PHPS film irradiated by 103 mW cm^-2^ VUV light. The inserted chemical structures are indicating the possible Si-N species which may generate from photo-dehydrogenation reaction.


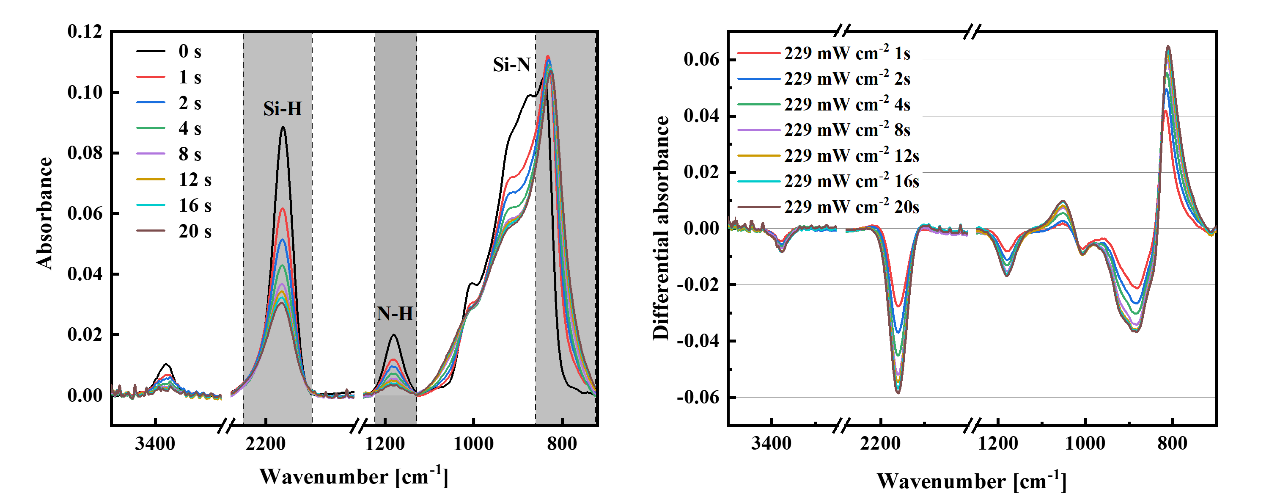


**Figure S2.** FTIR spectra changes (left) and differential spectra (right) of PHPS film irradiated by 229 mW cm^−2^ VUV light.


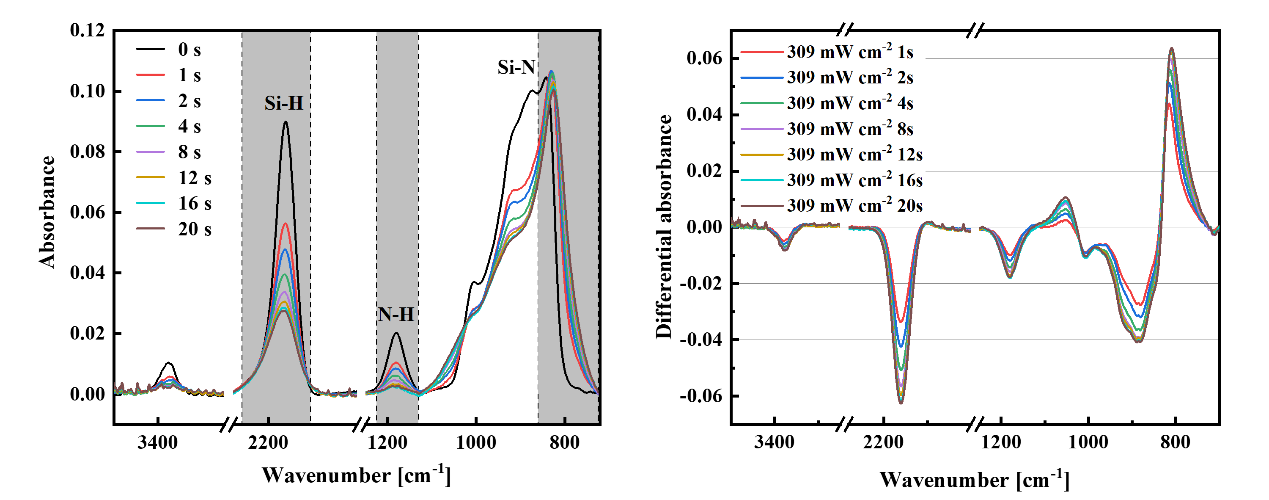


**Figure S3.** FTIR spectra changes (left) and differential spectra (right) of PHPS film irradiated by 309 mW cm^−2^ VUV light.

**
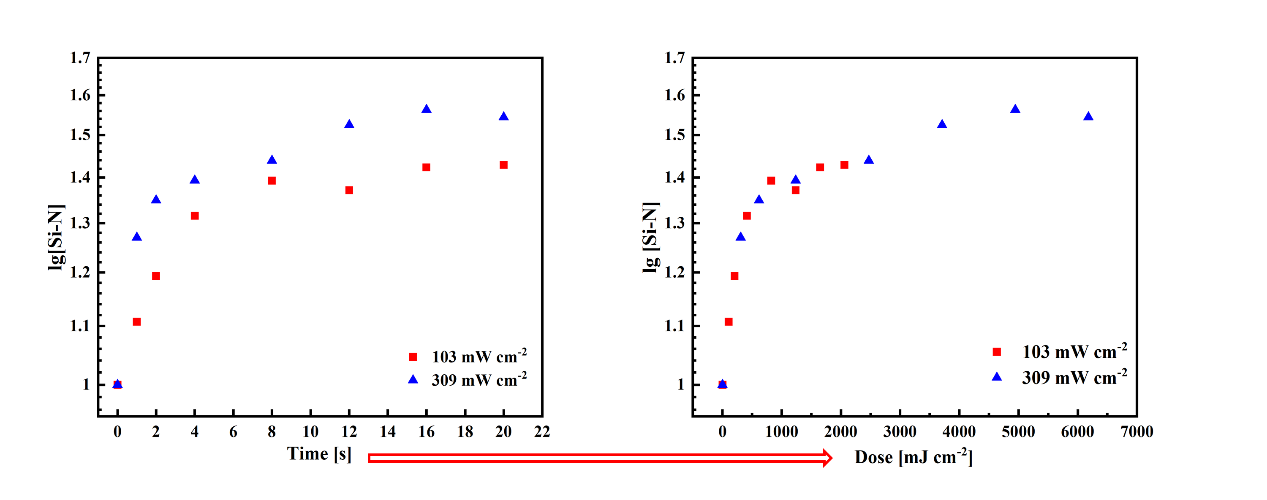
Figure S4.** Normalized change in Si-N bond ratio as a function of time (left) and dose (right) under different VUV light intensities.


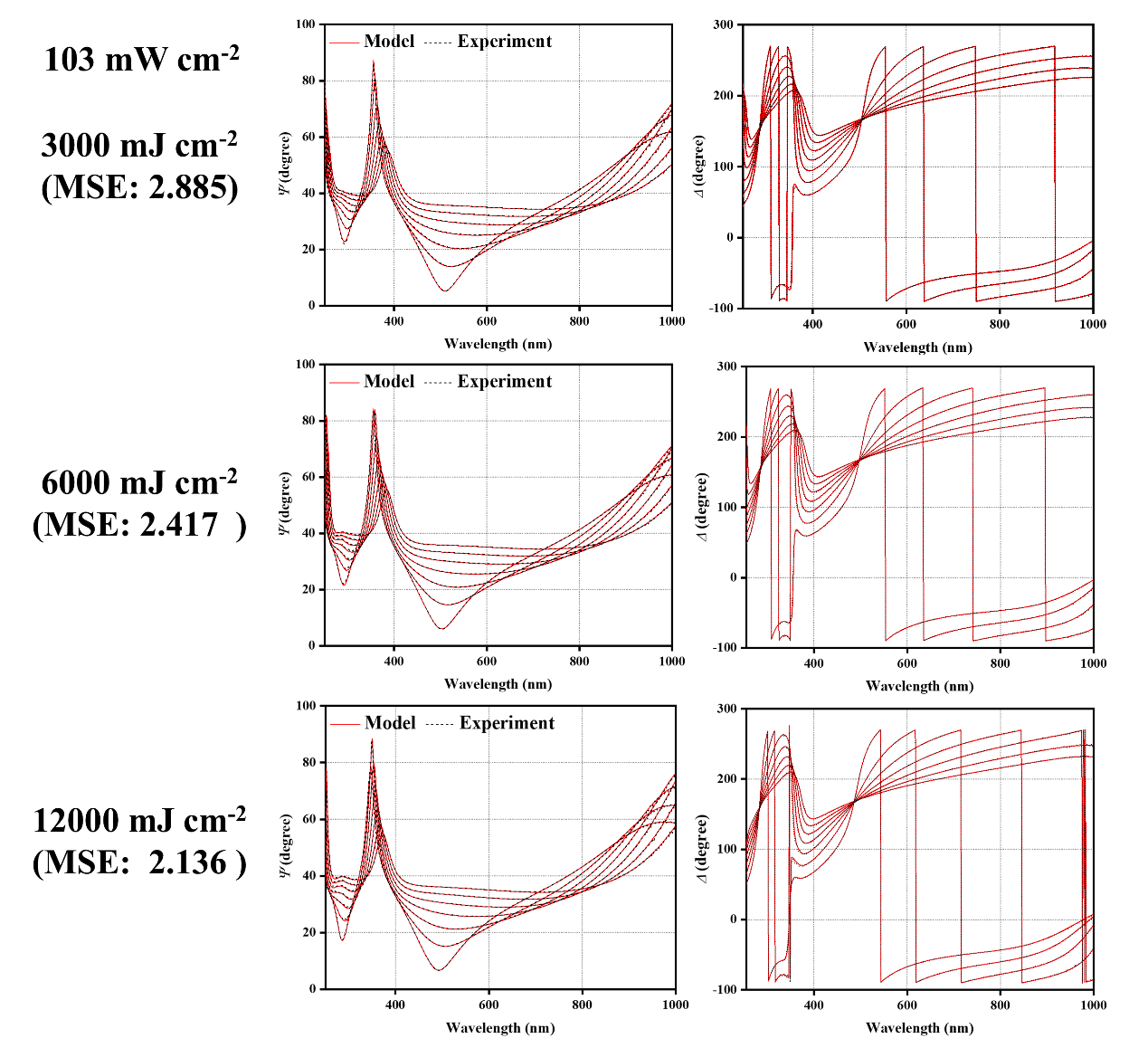


**Figure S5.** Experimental and fitting results (Ψ and Δ) of ellipsometry measurements for PHPS layers under 103 mW cm^−2^ VUV irradiation with the dose of 3000, 6000 and 12000 mJ cm^-2^.


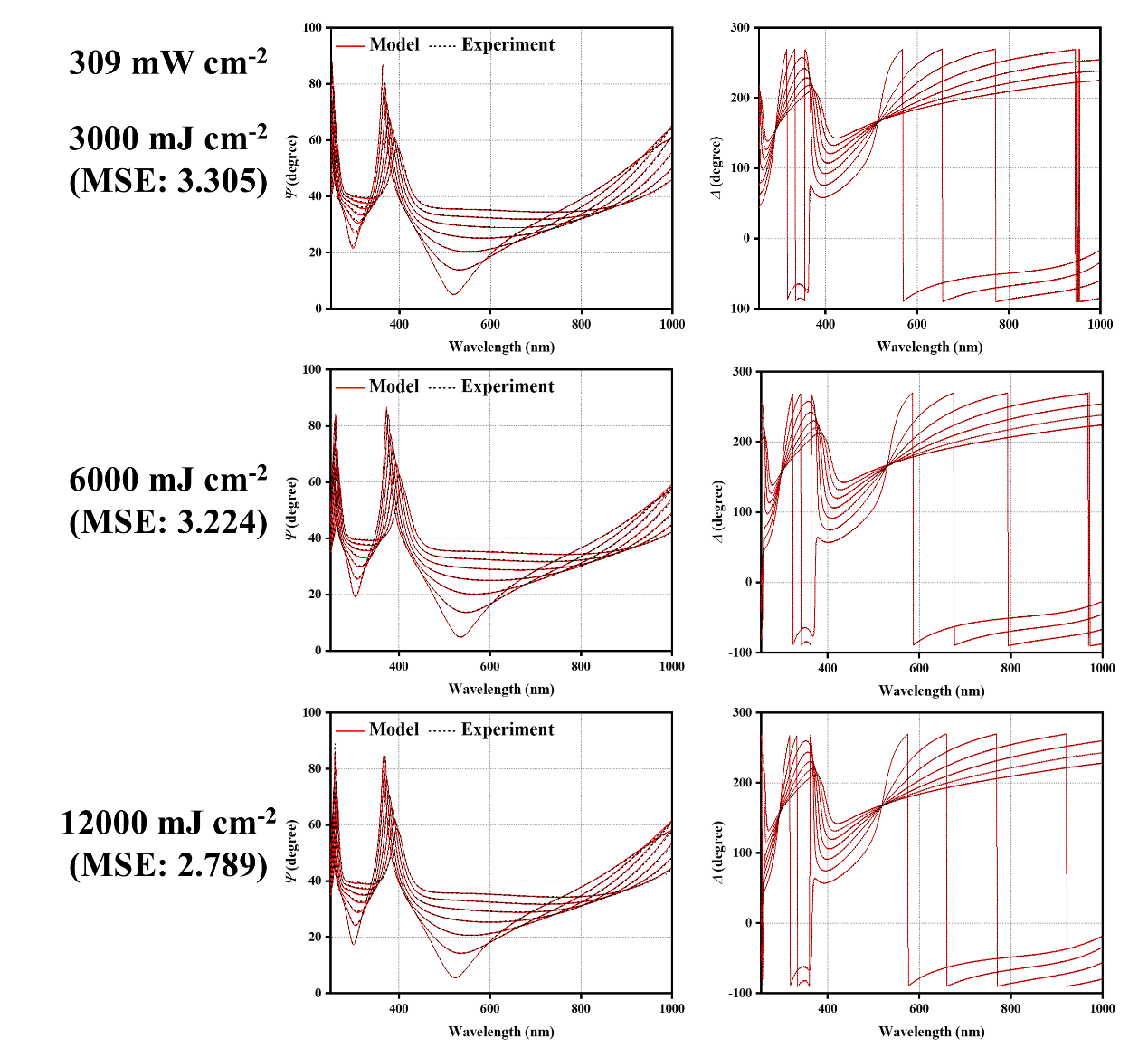


**Figure S6.** Experimental and fitting results (Ψ and Δ) of ellipsometry measurements for PHPS layers under 309 mW cm^-2^ VUV irradiation with the dose of 3000, 6000 and 12000 mJ cm^-2^.


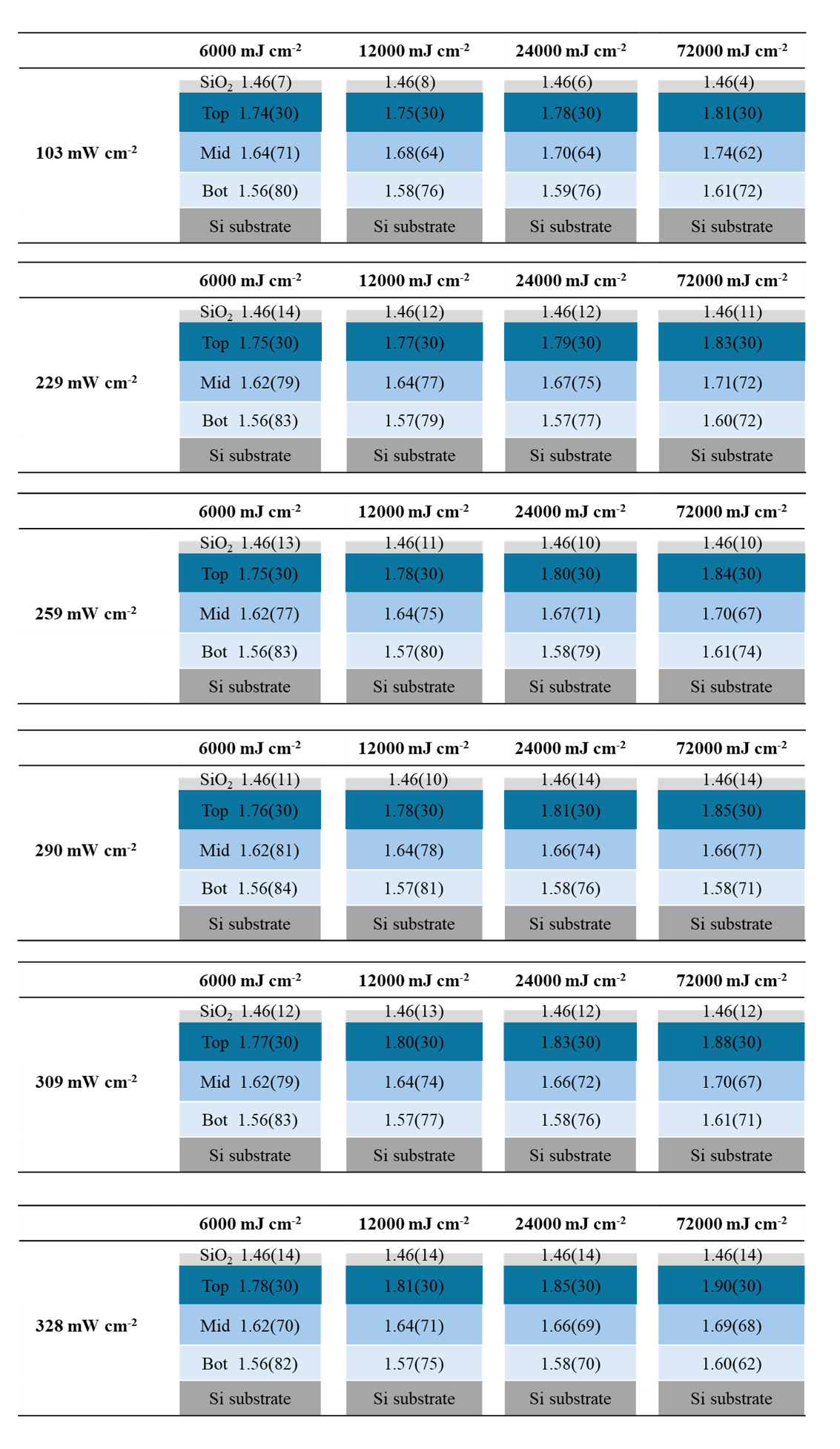


**Figure S7.** Refractive index distributions in the PDSN layers irradiated at 103 to 309 mW cm^-2^ with different doses.


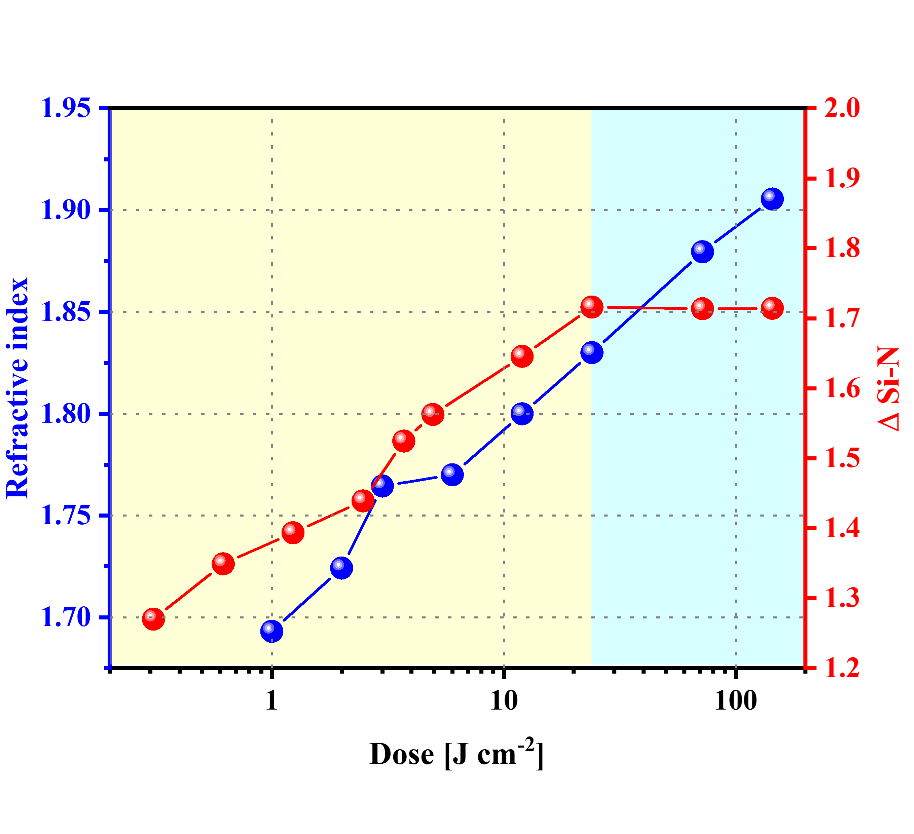


**Figure S8.** VUV irradiation dose dependence of the Si-N peak change and refractive index of Top30nm under 309 mW cm^-2^.


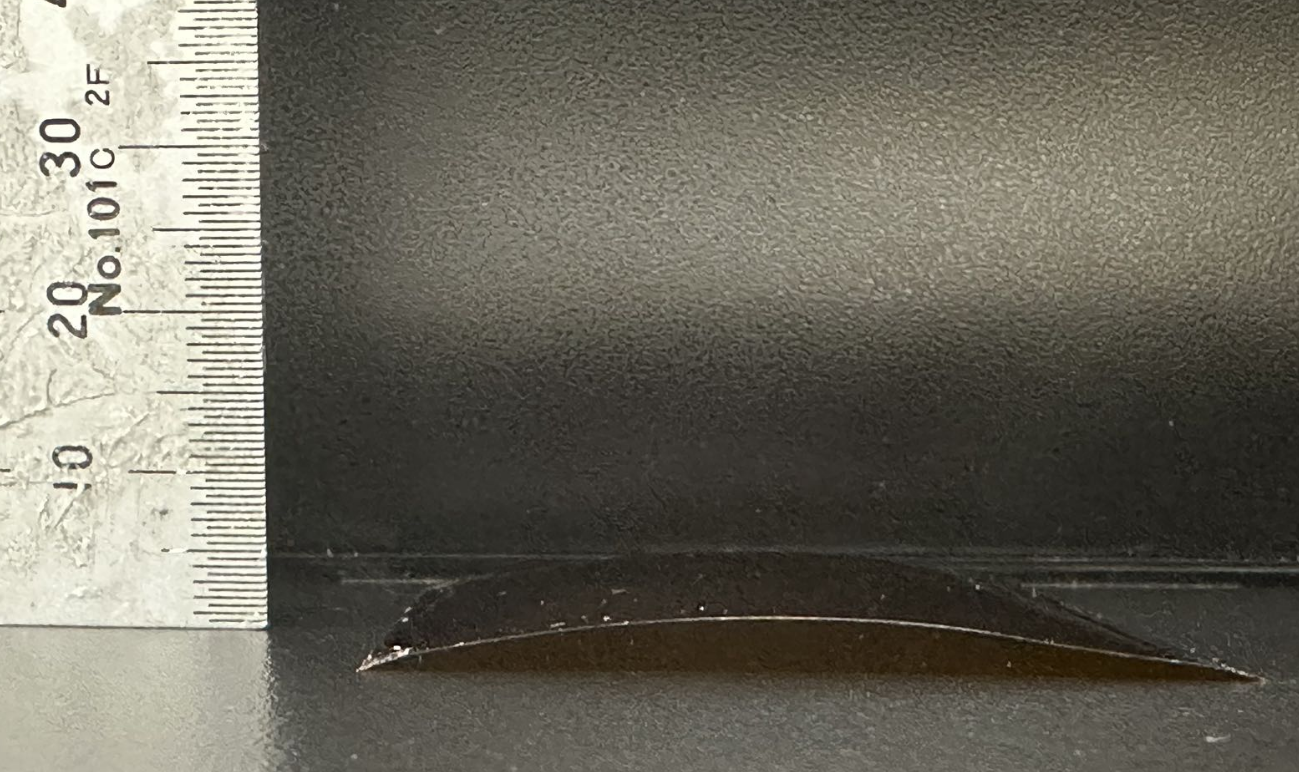


**Figure S9.** Picture of 3-unit barrier layer on top of PI film, showing minimal interfacial stress.


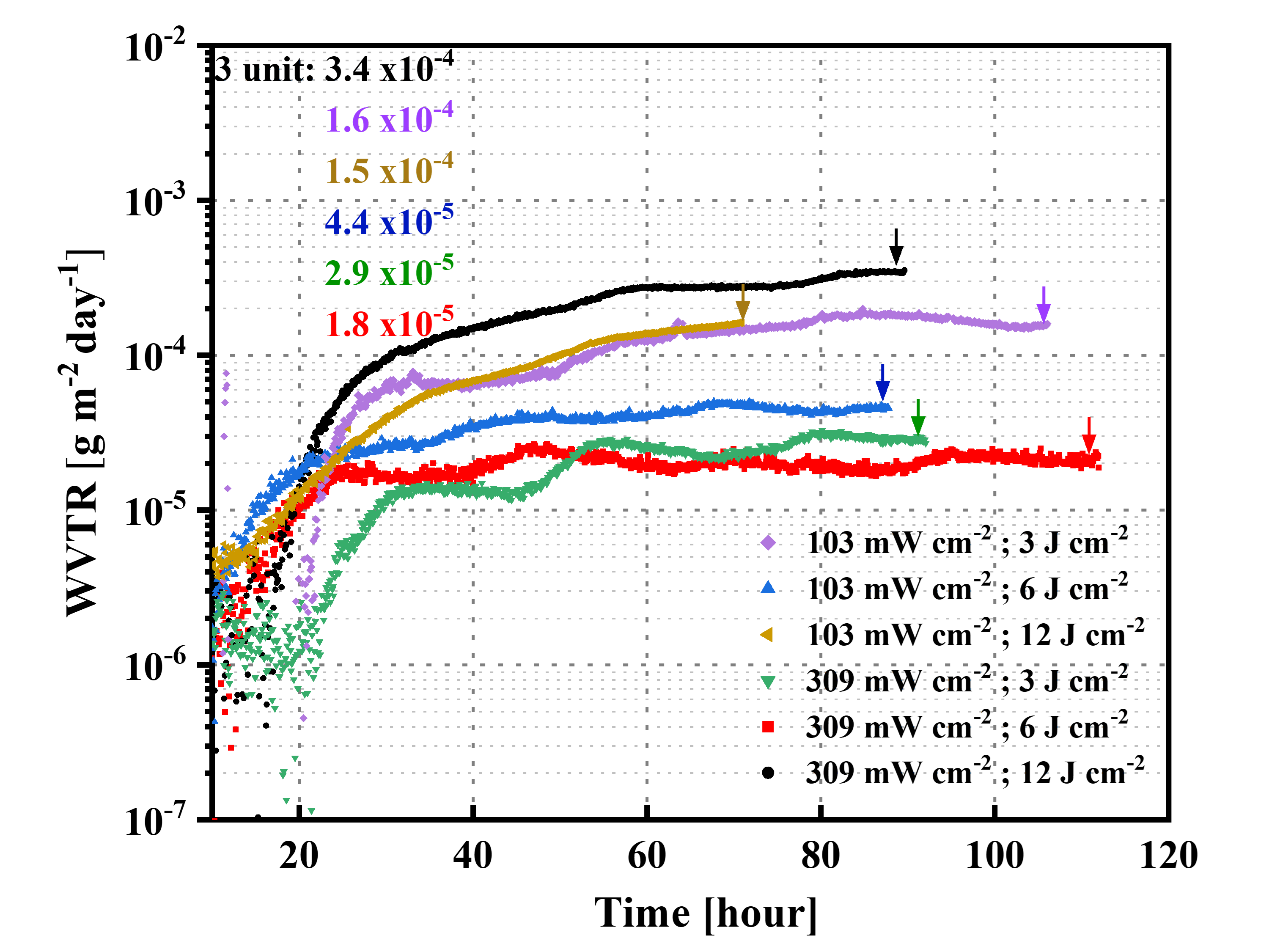


**Figure S10.** WVTR measurement data for barriers fabricated under different cumulative doses at 103 and 309 mW cm^-2^.

**Table S1**. Reported barrier performance using different materials produced in solution processes.

| Barrier type | Details | WVTR [g m^-2^ day^-1^] | Ref. |
| --- | --- | --- | --- |
| Organic polymer | BOPP film coating with PVOH/TEOS hybrid solution | 5.9 (37.8℃/90%RH) | ^[1]^ |
|  | CA/CNC-coated PP film | 1.3 (23℃/90%RH) | ^[2]^ |
|  | UV-curable polymer film | 0.031 (20℃/50%RH) | ^[3]^ |
| Organic-inorganic nano composite | cyclo-aliphatic epoxy/monodisperse silica nanoparticles /PET film | 0.24 (25℃, >90%RH) | ^[4]^ |
|  | Polyurethane/silicate /PET film | <0.05 (50%RH) | ^[5]^ |
|  | PVOH/clay nanosheet/PET film | 0.69 (23℃/90%RH) | ^[6]^ |
| PHPS-derived SiOx | SiOx/PET film | <10^-2^ (40°C/85%RH) | ^[7]^ |
|  | SiO_X_/CdSe/ZnS QD in PDMS/PEN film | 8.63×10^-3^ (37.8 °C/100 RH %) | ^[8]^ |
| PHPS-derived SiN | PET/PDMS/SiOx/SiNy/SiOxNy/PET film | <10^-4^ | ^[9]^ |
|  | PI/PDMS/PDSN/PDMS/PDSN/PDMS/PDSN/PI film | 4.8x10^-5^ (40℃/90%RH) | ^[10]^ |
|  | **PI/PDMS/PDSN/PDMS/PDSN/PDMS/PDSN/PI film** | **1.6×10^-5^**  **(40℃/90%RH)** | **this work** |

**Reference**

[1] A. Suhag, K. Biswas, S. Singh, A. Kulshreshtha, *Progress in Organic Coatings* **2022**, *163*, 106662.

[2] M. Nuruddin, D. M. Korani, H. Jo, R. A. Chowdhury, F. J. Montes, J. A. Howarter, J. P. Youngblood, *ACS Appl. Polym. Mater.* **2020**, *2*, 4405.

[3] D. Yang, Y. qiang Yang, Y. Duan, P. Chen, C. L. Zang, Y. Xie, D. M. Liu, X. Wang, Y. H. Duan, F. B. Sun, Q. Gao, K. W. Xue, *ECS Solid State Lett.* **2013**, *2*, R31.

[4] J. Jin, J. J. Lee, B.-S. Bae, S. J. Park, S. Yoo, K. Jung, *Organic Electronics* **2012**, *13*, 53.

[5] D. A. Kunz, J. Schmid, P. Feicht, J. Erath, A. Fery, J. Breu, *ACS Nano* **2013**, *7*, 4275.

[6] M. Röhrl, L. K. S. Federer, R. L. Timmins, S. Rosenfeldt, T. Dörres, C. Habel, J. Breu, *ACS Appl. Mater. Interfaces* **2021**, *13*, 48101.

[7] I. A. Channa, A. Distler, M. Zaiser, C. J. Brabec, H.-J. Egelhaaf, *Advanced Energy Materials* **2019**, *9*, 1900598.

[8] J. Kim, J. H. Jang, J.-H. Kim, K. Park, J. S. Jang, J. Park, N. Park, *ACS Appl. Energy Mater.* **2020**, *3*, 9257.

[9] L. Sun, K. Uemura, T. Takahashi, T. Yoshida, Y. Suzuri, *ACS Appl. Mater. Interfaces* **2019**, *11*, 43425.

[10] T. Sasaki, L. Sun, Y. Kurosawa, T. Takahashi, Y. Suzuri, *Advanced Materials Interfaces* **2022**, *9*, 2201517.
